# Toward Automatically Labeling Situations in Soccer

**DOI:** 10.3389/fspor.2021.725431

**Published:** 2021-11-03

**Authors:** Dennis Fassmeyer, Gabriel Anzer, Pascal Bauer, Ulf Brefeld

**Affiliations:** ^1^Machine Learning Group, Leuphana University of Lüneburg, Lüneburg, Germany; ^2^Department of Sport Psychology and Research Methods, Institute of Sports Science, University of Tübingen, Tübingen, Germany; ^3^Sportec Solutions AG, Subsidiary of the Deutsche Fußball Liga (DFL), Munich, Germany; ^4^DFB-Akademie, Deutscher Fußball-Bund e.V. (DFB), Frankfurt, Germany

**Keywords:** sports analytics, soccer, tracking data, variational autoencoders, labeling situations

## Abstract

We study the automatic annotation of situations in soccer games. At first sight, this translates nicely into a standard supervised learning problem. However, in a fully supervised setting, predictive accuracies are supposed to correlate positively with the amount of labeled situations: more labeled training data simply promise better performance. Unfortunately, non-trivially annotated situations in soccer games are scarce, expensive and almost always require human experts; a fully supervised approach appears infeasible. Hence, we split the problem into two parts and learn (i) a meaningful feature representation using variational autoencoders on unlabeled data at large scales and (ii) a large-margin classifier acting in this feature space but utilize only a few (manually) annotated examples of the situation of interest. We propose four different architectures of the variational autoencoder and empirically study the detection of corner kicks, crosses and counterattacks. We observe high predictive accuracies above 90% AUC irrespectively of the task.

## Introduction

The acquisition of tracking/positional and event data has become ubiquitous in professional football. The benefits of the resulting digital reproduction of a match, widely available in professional leagues, are twofold: Firstly, coaches, analysts and other decision makers in clubs may use data as an objective and quantitative alternative to traditional analyzes of performance, and, secondly, the collected data enables media to tell automated stories, to provide data-driven insights in what is happening on the pitch.

For example, match-analysis departments have historically spend vast amounts of time analyzing their upcoming opponent before each match by manually evaluating video footage. This work intensive approach is nowadays being supported or even partially replaced by automatic insight generation based on available data. While some information is easily accessible from the collected data, e.g., extracting the preferred formation of a team (Shaw and Glickman, 2019), other (rather tactical) pieces of information cannot be automatically computed yet, either because they are too complex (e.g., how teams behave during counterattacks), depend on the actual game philosophy of a team, require large amounts of tactical knowledge, or are considered a niche with only few interested followers. Detecting such events and patterns automatically offers a huge potential for performance analysis and may revolutionize current pre- and post-match performance analyses in professional football.

When speaking about data in soccer, we differentiate between positional/tracking and event data. Positional data, describing player and ball positions at any point in time of a match, are collected automatically via computer vision algorithms and dedicated tracking cameras. Event data, on the other hand, provides basic annotations of game events (mainly on ball actions like passes, shots, tackles, etc.) and is still acquired manually by human operators. The manual collection of such events is unsurprisingly labor and cost intensive and involves up to five operators per game. The goal of this article is to bridge the gap from the status quo toward fully-automatic annotations of soccer games.

There are several recent studies aiming to detect basic events directly out of video footage (Ekin et al., 2003; Wickramaratna et al., 2005; Kolekar and Palaniappan, 2009) or positional data (Zheng and Kudenko, 2010; Motoi et al., 2012; Richly et al., 2016; Stein et al., 2019) and others focus on the identification of sophisticated tactical patterns (Hobbs et al., 2018; Andrienko et al., 2019; Shaw and Sudarshan, 2020; Anzer et al., 2021; Bauer and Anzer, 2021). The proposed approaches provide useful solutions for their respective tasks. However, they are also restricted to either a particular data source or type of events or pattern that is to be detected; none of the above approaches offer an all-encompassing framework to deal with general detection problems.

A challenge for designing a general detector of game situations is the available data structure. While vast amounts of positional data of players and ball exist, collecting the associated labels of interest is an expensive endeavor and requires manual annotation by human experts. For example, counterattack detection first involves defining strict criteria and definitions of counterattacks before engaging in extensive search processes to annotate the matching game snippets. Consequently, it is vital to reliably extract the game situations with little external supervision. In that sense, classical supervised learning methods fail to be a viable candidate since the algorithms typically require large amounts of annotated data to achieve a good generalization error (Erhan et al., 2010). However, a strategy to mitigate the necessity of a large number of labels is to incorporate abundantly available *unlabeled data* into the training process. While there are many conceivable ways to operate within such a semi-supervised framework, we focus particularly on the variational autoencoder (VAE) (Kingma and Welling, 2013; Rezende et al., 2014) family of methods.

Variational autoencoders learn implicit low-dimensional feature representations for input data by jointly training a probabilistic encoder and decoder network. The idea is that the original observations can be reconstructed (approximately) from this lower-dimensional feature space. In fact, our semi-supervised strategy relies on inferring these semantically salient representations for annotated situations, hence reducing the need to solve a large supervised learning problem in feature space. Our instance of semi-supervised learning achieves a substantial increase in generalization ability in cases where only a few observed labels are available (Kingma et al., 2014). An essential contribution of this paper is to lift the underlying principles to spatiotemporal structures to capture the temporal and spatial dependencies of positional data. Existing body of research on extending VAEs to sequential data mainly focuses on the generative aspects of the models rather than on their potential benefits in the context of semi-supervised learning (Chung et al., 2015; Goyal et al., 2017).

In this paper, we propose novel VAE-based feature extraction methods. Starting from the vanilla VAE, we begin with proposing a rather straight forward generalization that can be applied to positional data. A second contribution incorporates existing auxiliary labels in the training process. The idea of the auxiliary labels is to foster discriminative causes of variation in the inferred latent feature representation. The main contribution however is the development of sequential counterparts of the two VAEs to match the spatiotemporal problem domain. After one of the VAEs has been trained using unlabeled or auxiliary labeled data, only a few of the feature representations, for which labels of interest exist, are fed into a support vector machine to train the final classifier. We empirically evaluate the effectiveness of our approach on three different detection tasks, involving the detection of cornerkicks, crosses (labels obtained from event data), and counterattacks (labels manually annotated by experts). We observe detection rates above 90% AUC for all tasks and discuss several findings on methodological issues derived from further experimentation.

The remainder is structured as follows. Section Problem Setting introduces the formal problem setting. The static and sequential models are presented in sections Static Models, Sequential Models, respectively. We report on our empirical findings in section Empirical Evaluation and provide a discussion in section Discussion. Section Related Work reviews related work and section Conclusion concludes.

## Problem Setting

Positional data from professional soccer is introduced as follows. Let A be the set of agents (i.e., players and ball) and T be the set of timesteps. For each element of the cartesian product A×T, whereabouts of all agents on the pitch in form of two-dimensional coordinates (*g, h*) ∈ ℝ^2^ are observed. It will be convenient to further divide the set of agents into three disjoint subsets, $A_{1}$, $A_{2}$, and $A_{3}$, corresponding to the players on teams 1, team 2, and the ball^1^, respectively.

Individual spatiotemporal movements of the agents allow to augment the positional data with additional pieces of information such as the (approximated) velocity of players $\left(\frac{dg}{dt},\frac{dh}{dt}\right)$. More precisely, linearized motion for agent a∈A is computed via


$$\left(\Delta g_{t}^{\left(a\right)},\Delta h_{t}^{\left(a\right)}\right)=\left(g_{t^{\prime }}^{\left(a\right)}-g_{t}^{\left(a\right)},h_{t^{\prime }}^{\left(a\right)}-h_{t}^{\left(a\right)}\right)$$

with *t*′ > *t* and $\left(\Delta g_{t}^{\left(a\right)},\Delta h_{t}^{\left(a\right)}\right)=\left(0,0\right)$ for the case of $t^{\prime }\notin T$, i.e., using a small time window between two consecutive frames. Further defining Y as an *auxiliary label space* that consists of inexpensive labels (e.g., provided by event data), we are given a subset of event annotations $T_{Y}\subset T$ s.t. $\left|T_{Y}|\ll |T\right|$, referred to as $y_{S}≔\left\{y_{t}:t\in T_{Y}\right\}$. We further denote $Y_{b}$ as the (binary) *target space* described by an action value of interest and a “no action” value with $T_{Y_{b}}\subset T$ ($\left|T_{Y_{b}}|\ll |T\right|$) defining the set $y_{B}:=\left\{y_{t}:t\in T_{Y_{b}}\right\}$^2^. We denote the composite of all pixel coordinates and velocity values of agents *a* at a certain timestep as $x_{t}:={\left\{\left(g_{t}^{\left(a\right)},h_{t}^{\left(a\right)},\Delta g_{t}^{\left(a\right)},\Delta h_{t}^{\left(a\right)}\right)\right\}}_{a\in A}$ and formulate our objective as quantifying the probability over $Y_{b}$ given the state representation ***x***_*t*_ for all t∈T.

An emerging issue is to find a pertinent representation of the described data for model training. A plain random concatenation of the agents' coordinates and velocities at time *t* is clearly inappropriate in the sense that divergent instantiations of agent orderings also translate into divergent representations for the exact same state. Accordingly, the function that tranforms instances of ${\left\{\left(g_{t}^{\left(a\right)},h_{t}^{\left(a\right)},\Delta g_{t}^{\left(a\right)},\Delta h_{t}^{\left(a\right)}\right)\right\}}_{a\in A}$ into an input representation of a neural network needs to be invariant under permutation of the agents. Since the locations of the agents are given as pixel coordinates, we choose to convert these coordinates into an image-based representation, resulting in a consistent representational structure across different game settings.

The mechanism for capturing position and motion information in a 3-dimensional image representation ***x***_*t*_ is based on the approach presented in Dick and Brefeld (2019). Here, the pitch size (105 × 68) defines the axes in the horizontal and vertical directions, with each channel of the tensor encoding a different subset of the available information. The first 3 channels capture positional information of $A_{1}$, $A_{2}$ and $A_{3}$ (in that very order) by assigning constant 1 s to the coordinates defined by $\left(g_{t}^{\left(a\right)},h_{t}^{\left(a\right)}\right)\;\forall a\in A$ and the corresponding channel. Since agent positions live in real-world coordinates, a transfer into image pixels requires a translation $\left(g_{t}^{\left(a\right)},h_{t}^{\left(a\right)}\right)+t$ with $t=\left(\frac{105}{2},\frac{68}{2}\right)$, effectively shifting the origin from the center of the image to the top left corner. The remaining channels track motion information, with velocity values acting as value assignments for the indices instead of constant 1 s. The speed values in *g* direction (Δ*g*_*t*_) is covered for $A_{1}$, $A_{2}$, and $A_{3}$ in channels 4, 6 and 8; the information in *h* direction (Δ*h*_*t*_) is handled by channels 5, 7 and 9. All other values in the resulting input representation $x_{t}\in {\mathbb{R}}^{105\times 68\times 9}$ are 0.

In summary, the final dataset representing a soccer game is a collection of tensor representations for each timestep $D=\left\{x_{1},..,x_{\left|T\right|}\right\}$ with additional label sets *y*_*S*_ (auxiliary labels) and *y*_*B*_ (target labels). The goal is to use the available evidence and auxiliary labels to construct detectors that work effectively to identify situations of interest defined in $Y_{b}$. To this end, we adopt a two-stage optimization procedure, which relies on the derivation of semantically meaningful feature representations. This instance of semi-supervised learning is advantageous in the present context because a large part of the model training is already accomplished independently of the specific game situation of interest. Consequently, the general detection design can be described based on the following stages:

1. The training of a VAE-based feature extraction module to transform the high-dimensional tensor data ***x***_*t*_ into a low-dimensional embedding space.
2. The training of a classifier using the derived embeddings and the available label information.

**Figure 1:**
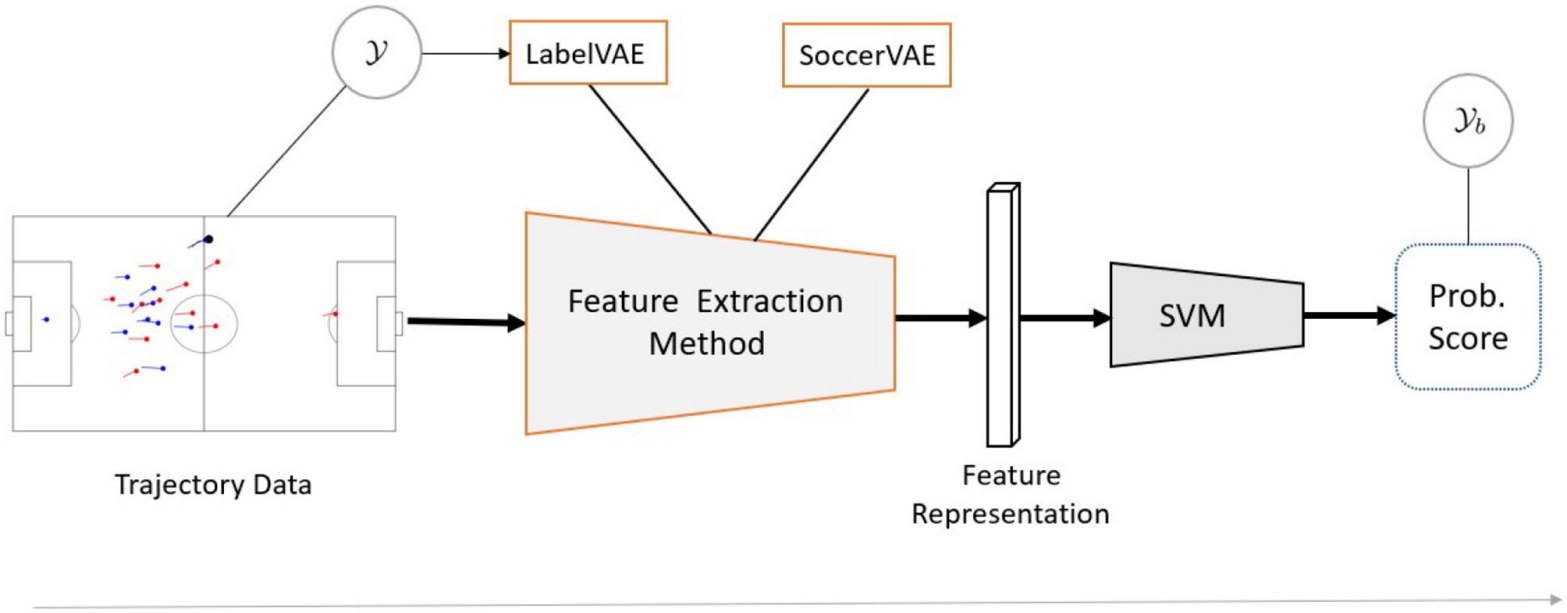
Detection worflow. The building blocks outlined in orange are presented in sections Static Models, Sequential Models and denote the technical contributions of this paper. The proposed LabelVAE methods for feature extraction operate on Y, while the SoccerVAE methods are trained fully unsupervised.

Irrespective of the first step's choice, we use a support-vector machine (SVM) (Cortes and Vapnik, 1995) for the second step. The technical contributions of this paper address the first stage and introduce novel feature extraction methods in sections Static Models and Sequential Models. See Figure 1 for an illustration of the information flow.

## Static Models

In this section, we present static models that operate only on a single timestamp to predict a labeling of the encoded situation. The term static stems from an equivalence class of model architectures whose resulting optimization targets are derived based on the assumption that each tensor frame is iid., i.e., the computation factors across the individual timesteps of a game. Note, however, that the data points themselves contain sequential information due to the inclusion of motion vectors for each agent. We discard the time subscripts for the tensor representations ***x*** since we operate within a static domain.

### Preliminaries

The idea of a variational autoencoder (VAE) (Kingma and Welling, 2013; Rezende et al., 2014) is to learn a deep generative model *p*_θ_(***x***, ***z***) = *p*(***z***)*p*_θ_(***x***|***z***) by maximizing the marginal log-likelihood of the training data D. Due to intractabilities that arise from the integration over the latent variables ***z***, the marginal likelihood is substituted by some variational lower bound to infer the model parameters. This requires introducing a variational approximation *q*_ϕ_(***z***|***x***), which is used to approximate the intractable true posterior. The resulting (negative) evidence lower bound (ELBO) denotes the VAE training criterion and enables concurrent optimization of θ and ϕ,


(1) $$\begin{array}{l} \log p_{\theta }\left(x\right)\geq {𝔼}_{q_{\phi }\left(z|x\right)}\left[\log p_{\theta }\left(x|z\right)\right]-\mathrm{KL}\left[q_{\phi }\left(z|x\right)∥p\left(z\right)\right]\\ \equiv -L_{\text{VAE}}\left(\theta ,\phi ;x\right). \end{array}$$

The first term of (1) quantifies the reconstruction error and the second term measures the distance between variational approximation and the pre-defined prior in terms of the KL divergence. The learned variational distributions *q*_ϕ_(***z***|***x***) capture semantically meaningful low-dimensional feature representations of the higher-dimensional observations ***x***. This encoded information facilitates finding a generalizable discriminator, especially when labels are scarce. The merits of such a semi-supervised instance are e.g., explored in the M1 model in Kingma et al. (2014), where samples from the approximate posterior distribution over the latent variables *q*_ϕ_(***z***|***x***) are used as input data for a downstream classifier (e.g., an SVM) to learn a decision boundary in latent space.

### SoccerVAE

We begin with a rather straight forward application of VAEs to the problem at-hand. The SoccerVAE uses the same optimization target as the vanilla VAE (cf. Equation 1) so that only the input and resulting choices on distribution type and architecture design need to be considered^3^. Regarding the former, the generating distribution of the generative model *p*_θ_(***x***, ***z***) is modeled as a multivariate distribution of independent Bernoulli parametrized by a decoder neural net with parameters θ:


$$\begin{array}{l} p_{\theta }\left(x|z\right)=\text{Bernoulli}\left(x|\mu \left(z;\theta \right)\right)=\overset{D}{\underset{j=1}{\prod }}\text{Bernoulli}\left(x_{j}|\mu_{j}\left(z;\theta \right)\right), \end{array}$$

where *D* is the dimensionality of ***x*** and $\mu {\left(\mu_{1},\ldots ,\mu_{D}\right)}^{⊤}$ aggregates the individual μ_*j*_ ∈ [0, 1] parameters for each pixel. This consitutes a reasonable design choice as we constrain the observed values to lie in the interval [0, 1].

Our generative and inference network definitions can be seen as instantiations of the class of CNN proposed by Radford et al. (2015). Specifically, the network **μ**(***z***; θ), which incrementally converts a sampled vector ***z*** to the observation space ***x*** ∈ ℝ^105×68×9^, is implemented using fractional-strided convolutions with ReLU activations (Nair and Hinton, 2010) and a sigmoid activation for the output layer, as well as batch normalization layers to reparametrize the intermediate layer activations (Ioffe and Szegedy, 2015; Bjorck et al., 2018). Each of the convolutional layers has kernels of the same size, with the number of kernels per layer decreasing proportionally to the depth of the network. All four proposed models deal with continuous priors given in form of standard multivariate Gaussians. The inference model *q*_ϕ_(***z***|***x***) is a diagonal Gaussian parametrized by an encoder neural net with parameters ϕ,


$$\begin{array}{l} q_{\phi }\left(z|x\right)=N\left(z|\mu \left(x;\phi \right),\text{diag}\left(\sigma^{2}\left(x;\phi \right)\right)\right). \end{array}$$

The role of the encoder is to transform a static game situations into fixed-size vector representations. We use strided convolutions with the leaky rectified activation (Maas et al., 2013; Xu et al., 2015) and batch normalization to process the input tensors. A fully-connected layer is dedicated to mapping the final representation onto the parameter space of *q*_ϕ_(***z***|***x***), i.e., to the mean and standard deviation vector of a diagonal Gaussian, which are used in conjunction with N(ϵ|0,I) to generate the latent vector ***z***.

### LabelVAE

The goal is to infer continuous latent embeddings that capture beneficial properties to detect a predefined (generally speaking: rarely occurring) game situation of interest. Hence, the quality of our approach is not primarily measured by reconstruction errors but in terms of the ability to discriminate between different types of situations in the subsequent supervised learning task. The second static model thus aims at directly optimizing a classification network. The model uses a VAE over the input variables that serves an effective regularizer. However, our envisaged optimization strategy is based on the extraction of general feature representations via pre-trained parameters to enable flexible adaption to the task at-hand.

The generative model reflects that causal factors of the observed ***x*** can be broadly categorized into label-specific and label-unspecific factors,


(2) $$\begin{array}{l} p_{\theta }\left(x,a,z\right)=p_{\theta }\left(x|a,z\right)p\left(z\right)p\left(a\right), \end{array}$$

where we assume that ***a*** encapsulates all relevant label-specific information and ***z*** the remaining label-unspecific characteristics. The dependency structure of the inference model embodies the consideration that the data-specific latent information ***z*** may vary with respect to the class-specific information of ***a***, that is,


(3) $$\begin{array}{l} q_{\phi }\left(a,z|x\right)=q_{\phi }\left(a|x\right)q_{\phi }\left(z|a,x\right). \end{array}$$

The above approximate posterior is amenable to approximating the true posterior over the latent variables to provide a tractable lower bound on the log-likelihood log*p*_θ_(***x***). The resulting (negative) ELBO is the optimization target of an unsupervised data point


(4) $$\begin{array}{l} \log p_{\theta }\left(x\right)=\log\int \int p_{\theta }\left(x,a,z\right)dzda \end{array}$$

(5) $$\begin{array}{l} \begin{array}{c} ={𝔼}_{q_{\phi }\left(a|x\right)}[{𝔼}_{q_{\phi }\left(z|x,a\right)}\left[-\log p_{\theta }\left(x|z,a\right)\right]\\ -\mathrm{KL}\left[q_{\phi }\left(z|x,a\right)∥p\left(z\right)\right]]-\mathrm{KL}\left[q_{\phi }\left(a|x\right)∥p\left(a\right)\right]\\ \equiv -L_{u}\left(\theta ,\phi ;x\right) \end{array} \end{array}$$

To encourage the model to capture the most relevant variational factors in the representations obtained via inference, we embed the available supervised learning signals concurrently with the unsupervised learning signals by means of an auxiliary classifier. Thus, the learning process is given by jointly maximizing the probability of each frame log*p*_θ_(***x***) and minimizing the auxiliary loss given the latent space realizations ***a***,


(6) $$\begin{array}{l} L_{s}\left(\theta ,\phi ,\xi ;x,y\right)=L_{u}\left(\theta ,\phi ;x\right)-\alpha {𝔼}_{q_{\phi }\left(a|x\right)}\left[\log q_{\xi }\left(y|a\right)\right], \end{array}$$

where ξ are the parameters of the classifier, α is a hyperparameter encoding the trade-off between generative and discrimative learning and *q*_ξ_(*y*|***a***) = Cat(*y*|**π**(***a***; ξ)). Equation (6) is essentially a regularized classification objective. More precisely, the second term quantifies the performance of a deep classification network with injected noise from the sampling operation ***a*** ~ *q*_ϕ_(***a***|***x***) and the variational loss $L_{u}$ can be viewed as a form of regularization imposed on the learned representations of the supervised prediction model.

The full training criterion is then given by collecting $L_{s}$ and $L_{u}$ for the supervised and unsupervised data points of the evidence D:


(7) $$\begin{array}{l} L_{LabelVAE}\left(\theta ,\phi ,\xi ;D_{u},D_{s}\right)=\underset{\left(x,y\right)\sim D_{s}}{\sum }L_{s}\left(\theta ,\phi ,\xi ;x,y\right)\\ +\gamma \underset{x\sim D_{u}}{\sum }L_{u}\left(\theta ,\phi ;x\right), \end{array}$$

where $D_{s}:=\left\{\left(x_{t},y_{t}\right),\forall t\in T_{Y}\right\}$ and $D_{u}:=D\backslash D_{s}$, and trade-off γ balances the contribution of the unsupervised term to the overall objective. This can be advantageous in situations where the labeled data is very sparse (*N*_*l*_≪*N*_*u*_) and therefore aim to externally impinge on the relative weight that is otherwise implicitly given by the data set (Siddharth et al., 2017). We define the feature vector for SVM training by concatenating the derived variables ***a*** and ***z*** into a single vector: [***a***, ***z***].

## Sequential Models

A clear limitation of the static models of the previous section is that their input is solely a single snapshot of the game. Although direction of movement and velocities may add context to the otherwise isolated situation, the idea of processing short sequences around these situations may add important information. Hence, in this section, we present sequential variants of the previously introduced models.

We denote a slice of consecutive frames from the game D as ***x***_≤*T*_, where *T* denotes the length of the game segment. Importantly, this implies that the time specifications of the frames ***x***_*t*_ refer more narrowly to the timestep in a segment within the soccer game ***x***_≤*T*_ = ***x***_1_, …, ***x***_*T*_ and no longer to the timestep in the overall game (as we describe it in section Problem Setting).

### SeqSoccerVAE

A viable avenue for inferring sequence-level features is to reconstruct the input sequence using a single global latent variable ***z***. While most approaches from the literature have been developed for modeling data distributions, we revisit this approach primarily to aggregate game sequences/multi-agent trajectories into informative vectors. Here we simply adapt the static VAE objective (1) to a sequential definition by assigning a temporal dimension to the data points:


(8) $$\begin{array}{l} {ℒ}_{\text{SeqSoccerVAE}}(\theta ,\phi ;x_{\leq T})={𝔼}_{q_{\phi }(z|x_{\leq T})}[\log p_{\theta }(x_{\leq T}|z)\\ \;-Kℒ[q_{\phi }(z|x_{\leq T})∥p(z)]. \end{array}$$

**Figure 2:**
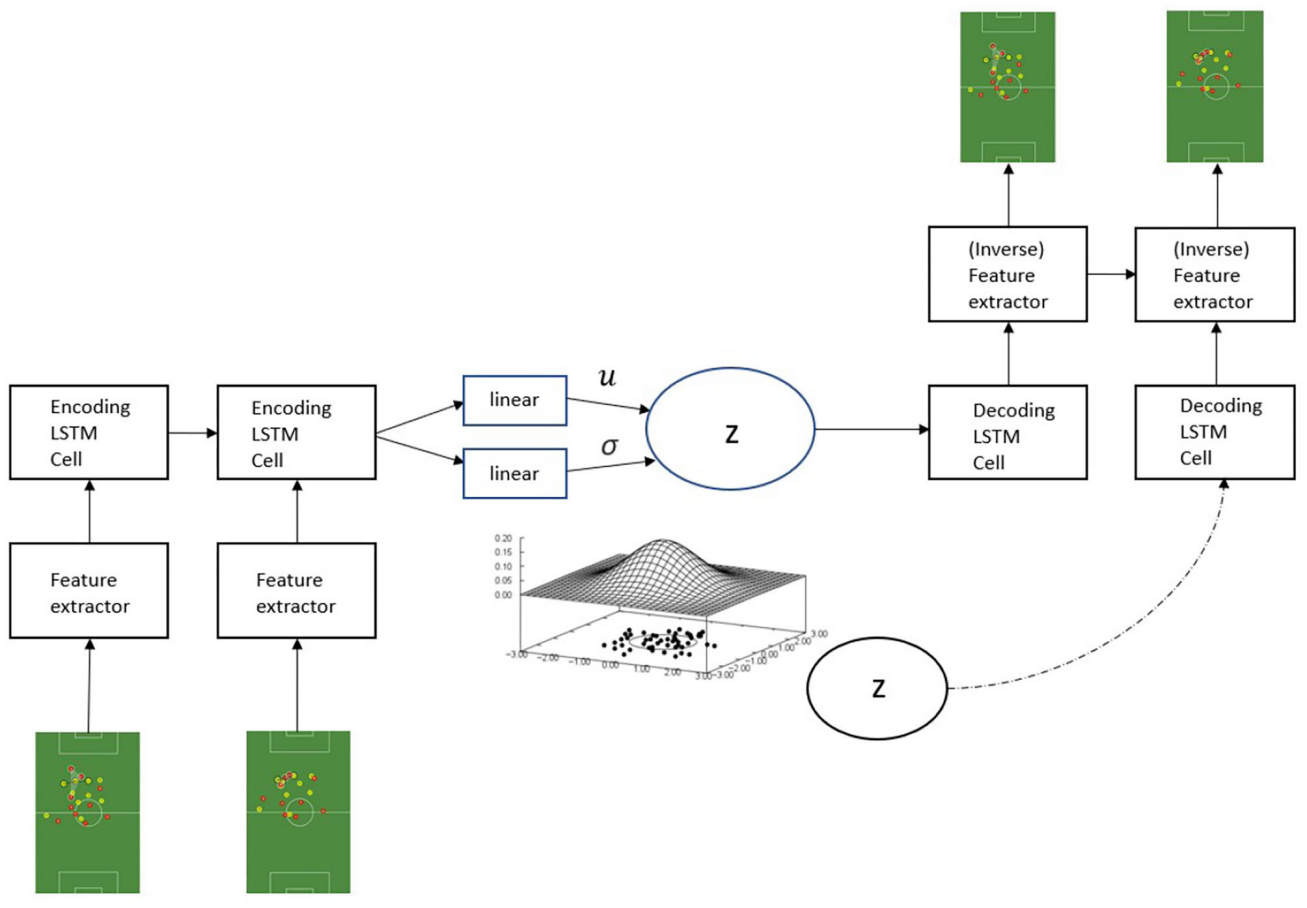
The SeqSoccerVAE. The model takes a sequence of frames as input and then extracts corresponding feature representations per timestep, which are passed to an LSTM network that outputs a global summary of the sequence. The sampled latent representation is used to reconstruct the sequence.

To model the components constituting Equation (8), we generalize the parameter functions for a given point to architectures suitable for sequential data. Accordingly, the parameters of the approximate posterior *q*_ϕ_(***z***|***x***_≤*T*_) are obtained from the last hidden state of an encoder RNN (parameterized by ϕ) working on the input sequence, and the generating distribution *p*_θ_(***x***_≤*T*_|***z***) is modeled by a decoder RNN (parameterized by θ) conditioned on the sampled hidden code alongside the previous data point, yielding the generating distribution $p_{\theta }\left(x_{\leq T}|z\right)=\prod_{t=1}^{T}p_{\theta }\left(x_{t}|z,x_{<t}\right)$. Thus, we force the model to encode all information about the data into the latent variable since it is the only source of information available for data reconstruction. The overall workflow of the SeqSoccerVAE is illustrated in Figure 2.

### SeqLabelVAE

The static LabelVAE in section LabelVAE seeks to leverage discriminative information already existing in the data by injecting them into the latent space via a classification network to facilitate the detection of game situations. In this section, we propose a sequential generalization of the LabelVAE that builds upon the dependencies in inference and generative parts of its peer. Accordingly, the SeqLabelVAE utilizes a label-specific partition of the latent space into ***a***_*t*_ and ***z***_*t*_, describing two distinct pieces of information about the data. We address the temporal dependency for successive observations by generating conditional independence for the random variables (the data and the latent variables) given the hidden states of two separate RNN networks,


$$\begin{array}{ll} h_{t}^{enc} & =f_{\phi }\left(x_{t},h_{t-1}^{enc}\right)\\ h_{t}^{dec} & =g_{\theta }\left(a_{t},z_{t},h_{t-1}^{dec}\right), \end{array}$$

where $h_{t}^{enc}$ denotes the recurrent state for the inference model and $h_{t}^{dec}$ denotes the recurrent state for the generative model.

The latent variables of the generative model at time *t* encode the observation ***x***_*t*_ indirectly via the state representation $h_{t}^{dec}$, yielding the conditional distribution *p*_θ_(***x***_*t*_|***z***_≤*t*_, ***a***_≤*t*_). As in the previous models, we restrict ourselves to standard multivariate Gaussian priors for both latent variables per timestep. Using unconditional prior distributions may reduce the approximability of observation sequences, but our focus is on obtaining informative feature representations rather than on generating sequences. For the inference model, we condition the LabelVAE dependency structure of the posterior approximation on the RNN state $h_{t}^{enc}$, resulting in the factorization


$$\begin{array}{ll} q_{\phi }\left(z_{\leq T},a_{\leq T}|x_{\leq T}\right) & =\overset{T}{\underset{t=1}{\prod }}q_{\phi }\left(z_{t}|a_{t},x_{\leq t}\right)q_{\phi }\left(a_{t}|x_{\leq t}\right). \end{array}$$

The derivations in the remainder of this section is analogous to the derivation of the static LabelVAE objective. Specifically, we optimize an unsupervised training instance by maximizing the ELBO


$$\begin{array}{c} J_{u}\left(\theta ,\phi ;x_{\leq T}\right)={𝔼}_{q_{\phi }\left(z_{\leq T},a_{\leq T}|x_{\leq T}\right)}[\overset{T}{\underset{t=1}{\sum }}-\log p_{\theta }\left(x_{t}|z_{\leq t},a_{\leq t}\right)\\ +\mathrm{KL}\left[q_{\phi }\left(z_{t}|x_{\leq t},a_{t}\right)∥p\left(z_{t}\right)\right]+\mathrm{KL}\left[q_{\phi }\left(a_{t}|x_{\leq t}\right)∥p\left(a_{t}\right)\right]]. \end{array}$$

Also, we enforce the latent variables to encode discriminative information by introducing an auxiliary classifier for the supervised training loss


**Figure 3:**
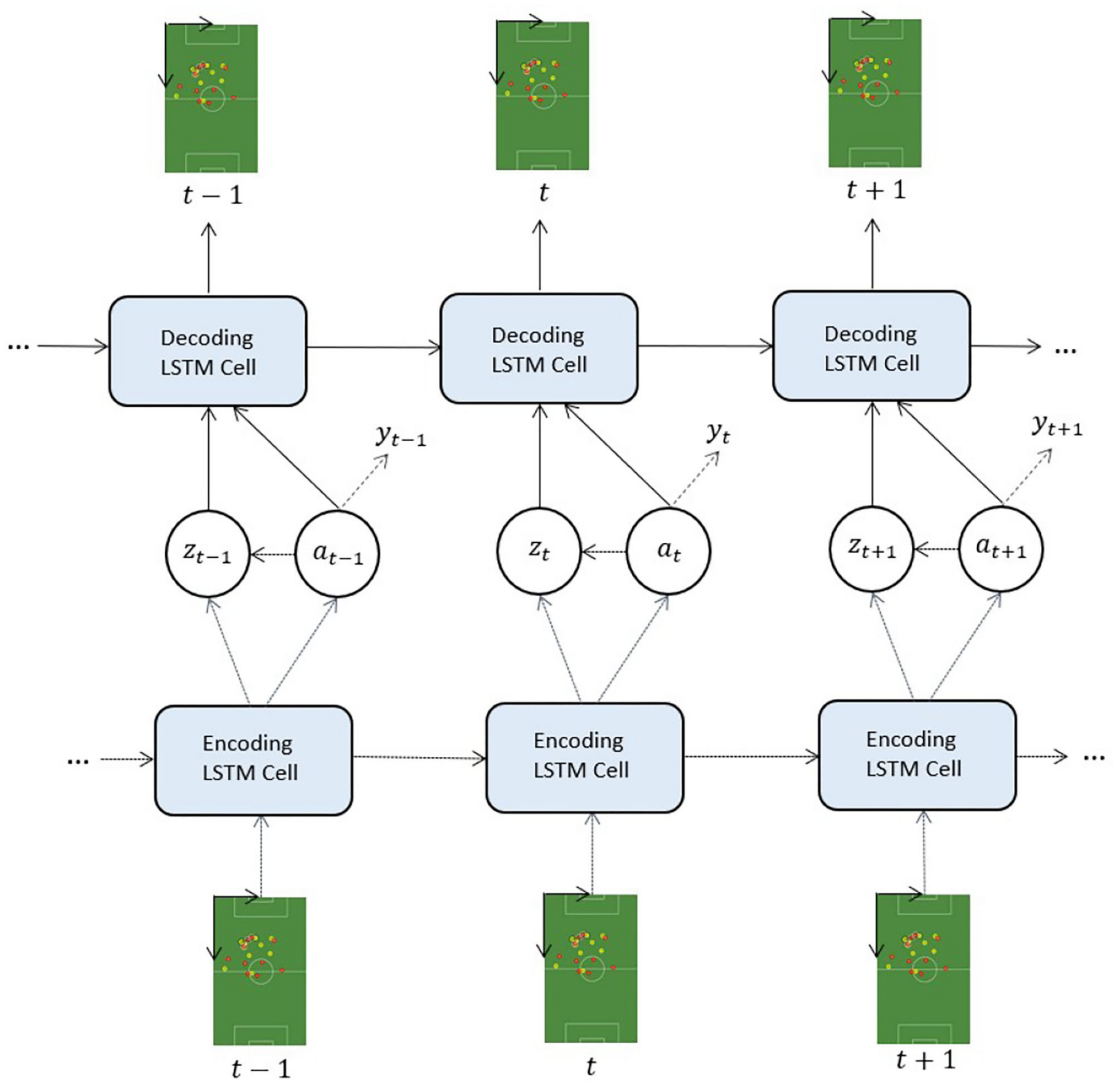
The SeqLabelVAE. Dashed lines indicate modules performing inference, solid lines denote components performing generation, and dotted lines point out the auxiliary classifier.

Js(θ,ϕ;x≤T,y)=Ju(θ,ϕ;x≤T)-α 𝔼qϕ(a≤T|x≤T)[∑t=1Tlogqξ(yt|a≤t)],

where log*q*_ξ_(*y*_*t*_|***a***_≤*t*_) is the per timestep classification loss and α is the hyperparameter that controls the trade-off between classification and generation. Note that the label y∈Y denotes the event annotation for the game situation ***x***_≤*T*_, such that each frame is assigned an identical label: *y*_1_ = … = *y*_*T*_ = *y*. We define the feature vector for classifier training on $Y_{b}$ by concatenating the derived variables ***a***_≤*T*_ and ***z***_≤*T*_ into a single vector: $\left[a_{1}^{\text{T}},...,a_{T}^{\text{T}},z_{1}^{\text{T}},...,z_{T}^{\text{T}}\right]$. The SeqLabelVAE architecture is sketched in Figure 3.

## Empirical Evaluation

### Data

We operate on two matches of the German national team. The tracking data consist of (*g, h*) positions of all players and ball, sampled at 25 frames per second. Following Dick et al. (2018), the tensor representations of the games are computed as follows. Firstly, the origin centered representation of the player position is transformed into pixel values of the tensor representation. This is done by adding half of the size of the pitch along the horizontal and vertical direction to the position of the agents. To approximate the velocities of the players and the ball at each timestep, we compute differences in positions over the last five frames (corresponding to a time lag of 0.2 s), yielding movement vectors of the form (Δ*g*_*t*_, Δ*h*_*t*_) = (*g*_*t*+5_−*g*_*t*_, *h*_*t*+5_ − *h*_*t*_). Since we assume the outputs to be Bernoulli distributed, we map the resulting speed values onto the range [0, 1]. To obtain the final input representation described in section Problem Setting, we incorporate the coordinates and velocity values into a 0-tensor of the size of the target shape (105, 68, 9). The updated tensor forms the input for a single timestamp. Every game consists of about 140, 000 such frame representations.

### Experimental Setup

As described in section Problem Setting, we define our setup with two different label spaces: the auxiliary label space Y that includes all available (inexpensive) labels and the binary (expensive) label space $Y_{b}$ that indicates occurrences of the game situation of interest. The auxiliary label space Y defines the label information *y*_*S*_ and originates in our study from the event data of the respective games (roughly 4000 observations per game). Note that only LabelVAE and SeqLabelVAE make use of these inexpensive labels in the training process to capture discriminative variations in the respective feature spaces. For simplicity, we focus on 5 auxiliary lables, Y = {*shot*, *cross*^^4^^, *ground*, *pass*, *other*}. If more than one auxiliary label is active in a snapshot, we select the minority label for the observations in question.

By contrast, the label space $Y_{b}$ defines the label information *y*_*B*_ used for SVM training and depends on the task at-hand. Our exemplary use cases target game actions of increasing difficulties by predicting variables encoded already in the available auxiliary labels or annotated manually by human experts. Accordingly, when employing fully unsupervised feature extraction methods (i.e., SoccerVAE and SeqSoccerVAE), targets *y*_*B*_ are the only label information required. We elaborate on the exact construction of the set *y*_*B*_ when discussing the predictive results in the following section.

We use one game for training and model selection and the other game for testing. In the training process, parameters of the static and sequential VAEs are optimized as well as parameters of the support vector machine which serves as the final classifier. After training, the best parameters are fixed and used for processing the test game. For every frame in the test game, probabilities of the quantities of interest are computed as follows: A static (section Static Models) or sequential (section Sequential Models) approach computes the embedding of the situation which is then used as input to the support vector machine which computes the prediction of interest and a softmax turns this prediction into a probability.

To assess the detection performance, we mainly use two different performance metrics: the area under the ROC curve (AUC) and the F1 score. We calculate the relevant components that constitute the F1 score (true positives (TPs), false positives (FPs), and false negatives (FNs)) as follows. To identify an action, we apply a threshold to the derived probability estimates for each frame of the test game. The independently detected frames are then converted into coherent game situations (or positive prediction instances), defined as a set of detected consecutive frames where the time gap between 2 successive frames is less than 10 s. The average length of the detected sequences depends on the concrete application, but it is in the range of a few seconds in most cases. We obtain TP values (FP values) if any (no) element within the extracted sequences is assigned the label of interest. Further, we define FN values as true action frames that remain undetected, i.e., do not occur within the positively predicted regions. We compute F1-scores for 50 distinct threshold values in the range between 0.6 and 0.98 and only report the maximum F1-score in the subsequent section^5^.

We compare our approaches to a fully supervised deep convolutional network that directly processes the tensor frames. The architecture of the baseline is identical to the feature extraction modules of our inference models, i.e., it consists of convolutional and batch normalization layers with LeakyReLu activation functions. The output dimensionality equals 1, and we use the standard binary cross-entropy loss for training. That is, the baseline directly computes the prediction of the desired label without a need for an additional SVM but lacks the reconstruction part of the proposed networks. We train the model with RMSprop (Tieleman and Hinton, 2012) and a batch size of 4. All methods are implemented with Tensoflow 2.0 (Abadi et al., 2016)^6^.

To ensure clarity regarding the used baseline architecture, we replaced “feature extraction modules of our inference models” with “encoder network of the SoccerVAE.” We report the comparison with this supervised baseline in Table 1.

**Table 1:** Results for the detection of cornerkicks, crosses and counterattacks.

**Task**	**Model**	**AUC**	**TP-Rate**	**Precision**	**F1**	**Length**
Cornerkick	Baseline	0.909	0.904	0.478	0.620	13.624
	SoccerVAE	0.944	0.940	0.578	0.716	14.445
	LabelVAE	0.967	0.877	0.670	0.760	8.451
	SeqSoccerVAE	0.975	0.886	0.792	0.824	11.054
	SeqLabelVAE	**0.986**	0.920	0.785	**0.850**	14.560
Cross	Baseline	0.827	0.765	0.507	0.606	20.070
	SoccerVAE	0.920	0.933	0.575	0.707	24.229
	LabelVAE	0.924	0.927	0.577	0.711	24.812
	SeqSoccerVAE	0.931	0.983	0.578	0.728	19.138
	SeqLabelVAE	**0.940**	0.812	0.683	**0.739**	16.750
Counterattack	SeqSoccerVAE	0.835	0.855	0.533	0.651	7.586
	SeqLabelVAE	**0.912**	0.745	0.726	**0.730**	3.712

*The highest values are indicated in bold face. The average length of the detected segments is given in seconds. All numbers are averages on the test game*.

### Predictive Accuracies

We showcase the expressivity of our approaches on three tasks with gradually increasing difficulty, the first one being the automatic **detection of cornerkicks**. The task should be the easiest one as the spatial distribution of agents is very indicative and event data provides ground-truth labels. The second task is the **detection of crosses**. Again, ground-truth is provided by event data, however, the spatial distribution of the agents is not as obvious as for cornerkicks. For both tasks, we train the models on one game and use another one for testing and evaluation.

The third task is the **detection of counterattacks** and clearly more involving than the former two. The task is more difficult than the previous two as many different temporal aspects need to be learned by the model, including gaining and maintaining ball possession, etc. Labels for this task are provided by human experts. Since the effort of labling is tedious, we train the models only on the first half of a game and evaluate on the second.

We begin with the detection of cornerkicks. For this straight forward task, the variational autoencoders are trained on a single game. The subsequent SVM is trained on 16 labeled examples per class (cornerkick vs. no cornerkick), where the negative examples are randomly drawn from the training game. The test game contains 26 cornerkick situations. The baseline uses the same training and testing set as the downstream SVM. Table 1 (top rows) summarizes the results for the different metrics on the test/validation game. All semi-supervised approaches outperform the fully-supervised baseline with SeqLabelVAE being the best predictor in this task. Comparing the static models shows decent improvements of the LabelVAE over the SoccerVAE. Furthermore, the average length of the detected sequences is significantly lower for the LabelVAE. Since the average length is a good indicator concerning the width of the predicted amplitudes, the value can be interpreted as a confidence measure of the predictions. Though LabelVAE performs worse than the sequential models, the static models provide solid results in this task, presumably because the agents' distribution on the playing field is easily distinguishable from other game situations. When comparing the sequential models, we find that the SeqLabelVAE performs better than SeqSoccerVAE. This improvement however comes at the cost of detection lengths.

Next, we study the detection of crosses using the same extracted features as for cornerkick detection. The classifier is trained on 33 examples per class (cross vs. no cross), and the test game consists of 38 cross situations. Table 1 (center rows) summarizes the results for the different metrics for the test/validation game. The trends are largely consistent with those of the corner detection task but at a lower overall level. The drop in performance stems from the variance in spatial distributions of agents that render the detection of crosses naturally more difficult than cornerkicks.

For the detection of counterattacks, static methods cannot sensibly be applied as the sequential nature and complexity of the situation (change of ball possession, maintaining ball possession thereafter, etc.) cannot be captured by focusing on only a single point in time. Consequentially, we only evaluate the sequential models using the first half of a manually annotated game with 27 counterattack situations for training and use the second half containing 33 situations for testing the classifier. The inherent complexity of counterattacks render the task much more challenging compared to the detection of cornerkicks or crosses. Table 1 (bottom rows) shows the results. As in the previous cases, the SeqLabelVAE emerges as the model of choice. Albeit detection performances are below previous ones, the findings show the potential of the models in challenging domains with manual labels. The detection rate of counterattacks is still above 91% AUC.

### Analyzing LabelVAE

**Figure 4:**
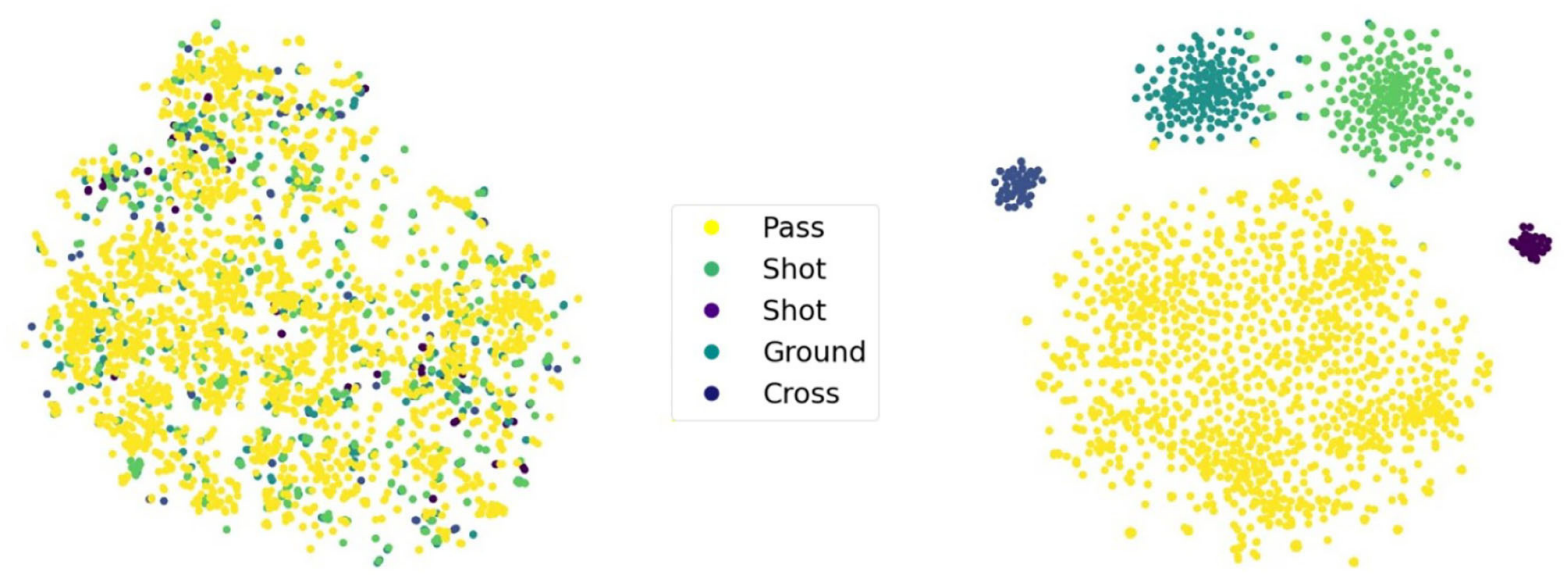
t-SNE visualizations of variable ***z*** (left) and variable ***a*** (right) of the LabelVAE. Each point in the plot is a tensor frame and the color represents a particular game action.

To shed light on the effect of the auxiliary labels used in LabelVAE and SeqLabelVAE, we visualize the latent space of the former using t-SNE (Van der Maaten and Hinton, 2008) in Figure 4. Recall that the generative model of LabelVAE makes use of two latent variables ***a*** and ***z***. The former encodes label-specific information while the latter captures all label-unspecific traits. Thus, both latent variables are supposed to capture different properties which actually holds true for the trained models as can be seen in the figure. Every point in the figure corresponds to a game situation and its color indicates the attached auxiliary label. The difference of the two latent variables is clearly visible and accentuated by a clear separation into action clusters (right part of figure) for ***a*** and the absence of any class structure (left part) for ***z***. Since both variables are used to reconstruct the tensor frames, but merely variable ***a*** concurrently needs to accurately discriminate between the different actions, it stands to reason that ***z*** captures position-specific information useful for frame reconstruction.

Recall, that the empirical results for the LabelVAE in Table 1 are based on concatenating the two latent variables ***a*** and ***z*** into a single feature vector yielding an AUC of 96.7% for cornerkicks. Passing on only a single variable to the SVM decreases the performance to 94.0% for ***z*** and 90.1% for ***a***, respectively. Hence, the two variables complement one another and focus on different aspects of the problem.

### Qualitative Assessment

**Figure 5:**
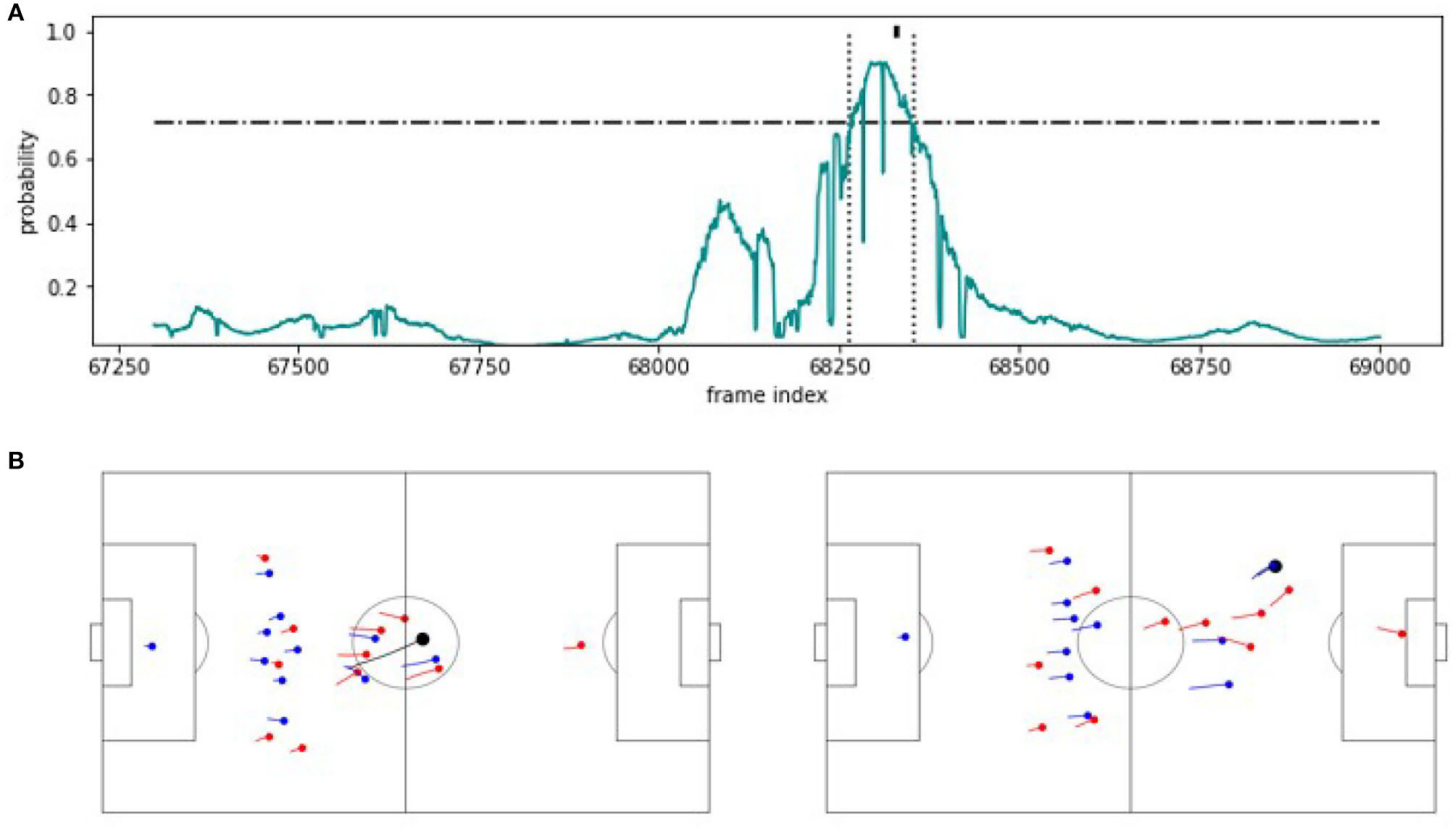
TP example. **(A)** Predicted probability values for an approximately 70 s excerpt from the validation game. The horizontal line indicates the threshold applied on the probabilities to recognize a counterattack. The vertical lines mark the beginning and the end of the extracted sequence. The mark at the probability value 1 indicates the counterattack annotation. **(B)** The corresponding snapshot visualizations for the beginning (left) and the end (right) of the detected scene.

To shed light on the nature of the proposed methodology, we compare the structure of correctly and incorrectly predicted examples for the detection of counterattacks on the example of SeqSoccerVAE. We begin with a correctly identified counter attack in Figure 5. The upper part of the figure shows the detection probabilities computed from the output of the SVM. The black indicator on top of the figure at timestamp 68.330 indicates the true label by the experts. The SeqSoccerVAE classifies the indicated segment above the threshold (dashed line) between timestamps 68.265 and 68.355 as a successful counterattack. The two figures below display the snapshots at the beginning and end of the detected scene and clearly show the successful counterattack that over both halves of halfes of the pitch.

**Figure 6:**
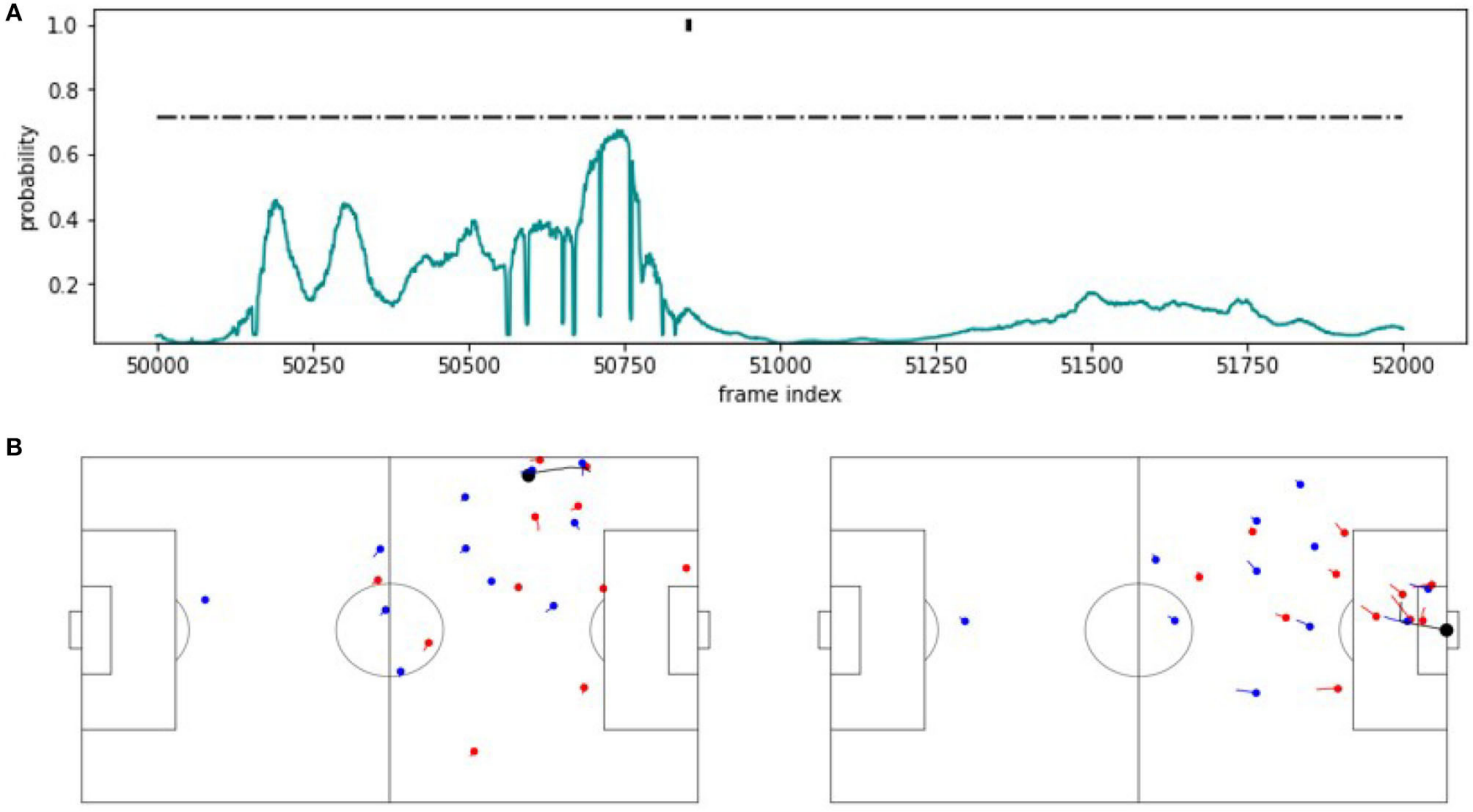
FN example. **(A)** Predicted probability values for an approximately 80 s excerpt from the validation game. The horizontal line indicates the threshold applied on the probabilities to recognize a counterattack. The mark at the probability value 1 indicates the corner annotation. **(B)** Snapshot visualizations of a potential start and end point around the counterattack annotation.

By contrast, Figure 6 shows a false negative. The detection probabilities shown in the upper part of the figure stay constantly below the threshold and consequentially, the turnover is missed by the classifier. Interestingly, the expert annotation is at a position, where the probability for a counterattack has decreased entirely and stays around zero. We credit this poor performance to the rather crowded origin of the situation and the many defending players behind the ball. The situation is clearly different from the one shown in Figure 5.

**Figure 7:**
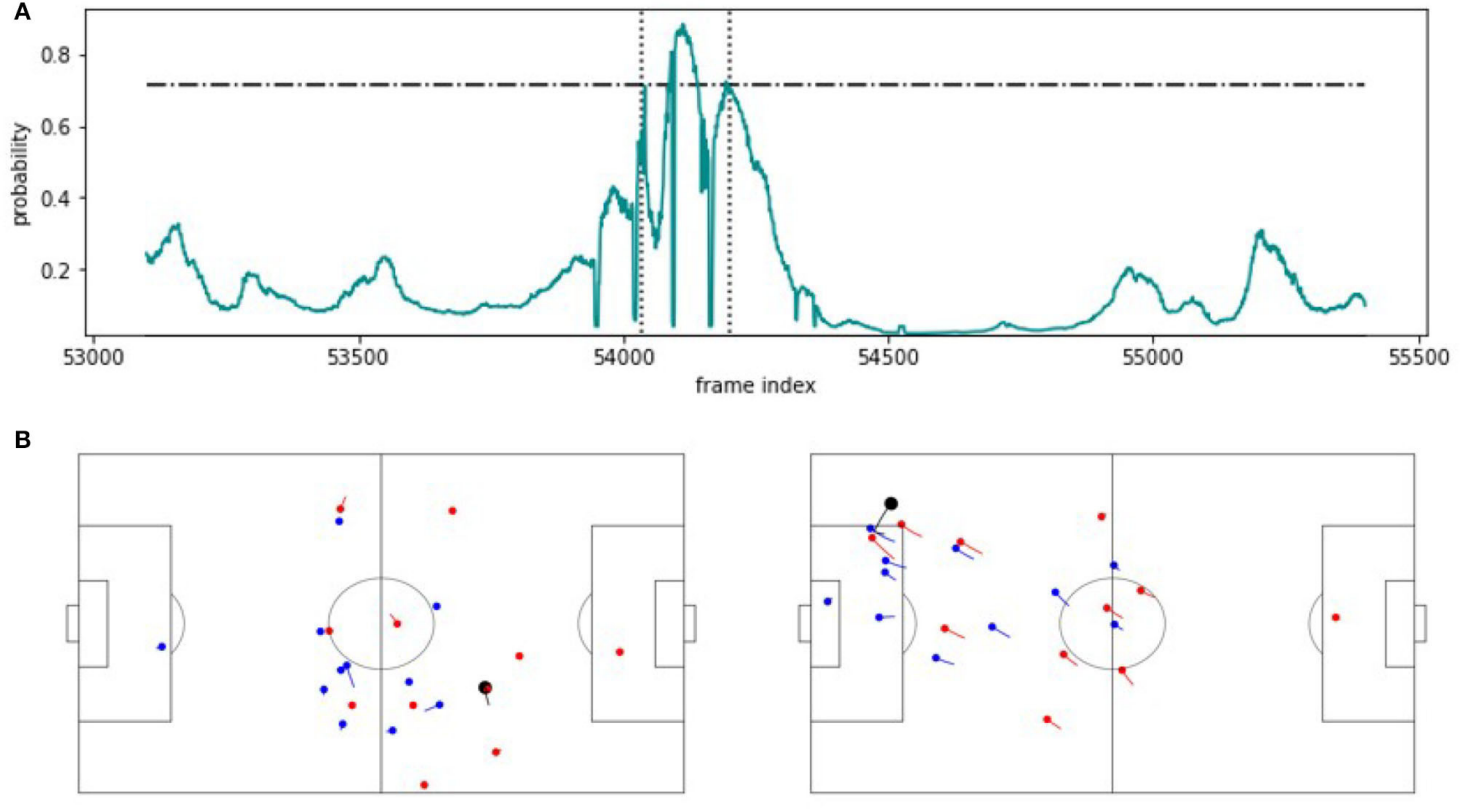
FP example. **(A)** Predicted probability values for an approximately 100 s excerpt from the validation game. The horizontal line indicates the threshold applied on the probabilities to recognize a counterattack. The vertical lines mark the beginning and the end of the extracted sequence. **(B)** The corresponding snapshot visualizations for the beginning (left) and the end (right) of the detected scene.

Last but not least, Figure 7 shows a false positive. As can be seen, the situation resembles the one agent in Figure 5 but here, the turnover fails and correspondingly, there is no expert annotation. This result expresses both, the strength and the limitation of the SeqSoccerVAE, and possibly the use of VAEs in general for such tasks. By using an autoencoder, we implicitly assume that similar situations in feature space will have a similar outcome in the real world. On one hand, this assumption allows to use many unlabeled situations to extract meaningful features and render the entire classification approach with only a handful of (expert) labels feasible. On the other hand, once the feature representation is fixed, the subsequent SVM is unlikely to differentiate neighboring situations although their labels suggest separation. However, the overall performance impressively shows that the latter case does not occur very often, resulting in an excellent total detection rate.

### Importance of Labeled Data

The idea of the paper grounds on splitting the original problem of labeling situations in soccer into two: an unlabeled^7^ grouping of similar situation by a variational autencoder (VAE) and feeding the learned feature representation into a support vector machine (SVM) to compute the final prediction. This approach promises a much better structured feature space that allows the SVM to learn an accurate hyperplane with only a few labeled instances. This, in turn, renders the approach useful for practitioners as they only need to provide manual labels for a handful of situations.

**Figure 8:**
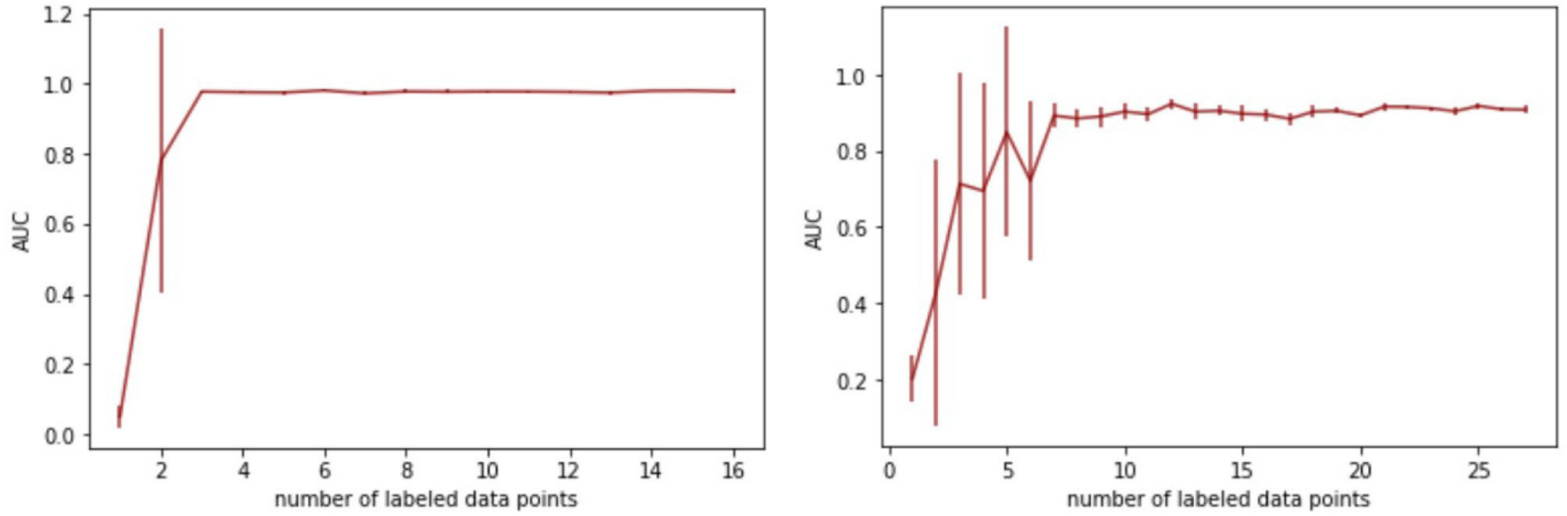
Resulting AUC scores for different training set sizes of the SVM. Left: SoccerVAE for cornerkicks. Right: SeqLabelVAE for counterattacks.

To investigate the models' applicability in a practical context, we quantify the (human) labeling effort to achieve accurate performance for the detection of cornerkicks and counterattacks, respectively. Figure 8 shows the results. The *y*-axis shows AUCs and the *x*-axis depicts the number of positive training examples which are (manually) labeled. In addition, the same amount of negative examples are introduced, however, these are randomly drawn from the training games and do not need manual attention. To reduce the effect of the randomness in the training sets, we report on averages over five runs; error bars indicate standard error. The left part of the figure shows the results for the SoccerVAE and the detection of cornerkicks. A training set with only six instances, three (manually) labeled positive and three randomly drawn negative ones, is sufficient to obtain optimal performance. Adding more instances to the training set does not lead to further improvements.

For the detection of counterattacks with the SeqLabelVAE (right part of figure), the performance stabilizes for about seven manually labeled data points. Increasing the size of the training set further reduces the variance that is introduced by selecting only a few positive and negative examples and renders the classifier more robust. However, the key message is that only seven manual annotations suffice to accurately detect counterattacks with a detection rate (AUC) of over 90%.

## Discussion

Our approach allows us to detect basic events (cornerkicks and passes) as well as more complicated patterns (i.e., counterattacks) without requiring massive sets of annotated data and without falling back to rule-based approaches. The detection of a more complicated pattern, namely counterattracks, is addressed in Hobbs et al. (2018) using an unsupervised clustering. By making use of a few expert-labels, we combine a data-driven approach with expert guidance. The autoencoder-based approach introduced by of Karun Singh^8^ is improved in two ways: First, we use a variational autoencoder and second, we extend the approach to use time series instead of static snippets of positional data. Bauer and Anzer (2021) compare a rule based model, to a machine learning based one to identify the tactical pattern of counterpressing automatically across 20, 000 labels from 97 matches. For their trained model they extract 137 hand-crafted features. The advantage of our approach is that it not only requires far fewer labeled observations, but also works with very simple basic features. It is easily reproducible for any other pattern, and, can be adjusted quickly even if definitions of patterns slightly change when the game-philosophy shifts (e.g., because of a coaching change).

Besides the potential to reduce the costs of manual event data collection, our approach enables several team performance affecting applications: The automatic detection of relevant patterns saves coaches and match-analysis departments not only time, but furthermore increases consistency and offers scalability. This can consequently be used to perform long-term analysis across multiple seasons or even leagues. Furthermore, besides match-analysis this methodology could also be integrated in the player scouting process, by identifying certain beneficial individual action patterns and finding players that exhibit these frequently.

While our work describes the technical framework to achieve these results, for it to be usable in a club environment, one would need to integrate it in an application that fits in seamlessly into daily routines of match-analysis or scouting departments.

## Related Work

This paper explores issues related to VAE-based semi-supervised learning, with the main contribution in this field introduced by Kingma et al. (2014). Our SoccerVAE and LabelVAE are clearly inspired by their proposals M1 and M2. Specifically, the authors integrate label information into the assumption of the data generation process, thereby obviating the necessity for the otherwise required supervised learning task on extracted label-feature pairs. Recent work by Joy et al. (2020) argues that explicitly modeling the connection between labels and their corresponding latent variables improves the classification accuracy compared to the M2 approach and allows to learn meaningful representations of data effectively. Maaløe et al. (2016) also improve M2 classification performance by introducing an auxiliary variable that leaves the original model unchanged but increases the flexibility of the variational posterior. This can result in convergence to a parameter configuration that is closer to a local optimum of the actual data likelihood (due to potentially better fits to the complex posterior) while maintaining the computational efficiency of fully factorized models. Siddharth et al. (2017) choose a more generalized formulation of semi-supervised learning with VAE compared to the models in the work by Kingma et al. (2014). Their framework allows choosing complex models, such as when a random variable determines the number of latent variables itself.

In addition to static semi-supervised tasks, this work methodologically touches a branch of research that describes methods involving autoencoders to model sequential data. Bayer and Osendorfer (2014) incorporate stochasticity into vanilla RNNs by making the independently sampled latent variables an additional input at each timestep. Chung et al. (2015) apply a similar model termed VRNN to speech data, sharing parameters between the RNNs for the generative model and the inference network. In Goyal et al. (2017), the latent variable participates to the prediction of the next timestep, and the variational posterior is informed about the whole future in the sequence modeled by an RNN processing the sequence backwards. While the previously mentioned methods sample a separate latent variable at each timestep, Bowman et al. (2015) propose an RNN-based VAE to derive global latent representations for sentences. The approach to modeling human-drawn images discussed in Ha and Eck (2017) shares many architectural similarities to Bowman et al. (2015), but uses an additional backward RNN encoder. Teng et al. (2020) introduce a semi-supervised training objective for modeling sequential data where the model specification draws inspiration from Kingma et al. (2014) and Chung et al. (2015).

## Conclusions

We studied automatic annotation of non-trivial situations in soccer. We proposed to separate the problem into an unsupervised autoencoder to learn a meaningful feature representation and a supervised large-margin classification. The advantage of this separation lied in the use of abundant unlabeled data that allowed for learning a nicely structured feature space so that only a few labeled examples were needed in the classifier to learn the target concept of interest.

We proposed two variants of autoencoders, a straight forward application of existing results (SoccerVAE) and a more sophisticated variant that used auxiliary labels and allowed for even more discriminative feature spaces (LabelVAE). In addition to these two static variants, we devised their sequential peers to account for the spatiotemporal nature of soccer. Empirically, we studied the performance of the four approaches on three different detection tasks, involving cornerkicks, crosses, and counterattacks. The SeqLabelVAE turned out the best competitor and outperformed all others with detection rates of 91% AUC or higher in all problems for only a few labeled examples.

While our methods emerged as valuable tools for detection tasks in soccer, there are some shortcomings that could be addressed in future work. A possible starting point is to compare the implicit regularization of our semi-supervised approach against supervised sequential models with alternate regularization methods (Semeniuta et al., 2016). From the perspective of achieving the lowest possible generalization error, there are several avenues for potential variations. Future work might include alternate probabilistic assumptions (Goyal et al., 2017; Joy et al., 2020) such as conditioning the variational distribution on the full input sequence (Goyal et al., 2017), novel regularization techniques for VAE (Tolstikhin et al., 2017; Ma et al., 2019; Deasy et al., 2020), other approaches to semi-supervised learning (Kingma et al., 2014; Dai and Le, 2015) such as transfer learning (Fabius and Van Amersfoort, 2014; Srivastava et al., 2015), or to achieving consistent agent representations such as graph-networks (Sun et al., 2019; Yeh et al., 2019) and tree-based role alignments (Lucey et al., 2013; Sha et al., 2017; Felsen et al., 2018).

## Data Availability Statement

The original contributions presented in the study are included in the article/supplementary materials, further inquiries can be directed to the corresponding author/s.

## Author Contributions

All authors listed have made a substantial, direct and intellectual contribution to the work, and approved it for publication.

## Conflict of Interest

The authors declare that the research was conducted in the absence of any commercial or financial relationships that could be construed as a potential conflict of interest.

## Publisher's Note

All claims expressed in this article are solely those of the authors and do not necessarily represent those of their affiliated organizations, or those of the publisher, the editors and the reviewers. Any product that may be evaluated in this article, or claim that may be made by its manufacturer, is not guaranteed or endorsed by the publisher.

## Footnotes

^1^We have $A_{i}\subset A$ s.t. $A_{1}\cup A_{2}\cup A_{3}=A$ and $A_{1}\cap A_{2}\cap A_{3}=\emptyset$.

^2^A description of the exact form and type of the label information used in this work is given in section Experimental Setup.

^3^Unless explicitly stated, these choices are reused in the derivation of the other models.

^4^The auxiliary label cross also includes corners and freekicks.

^5^Therefore, unlike the reported AUC values, the F1 scores are validation values as we engage in threshold optimization.

^6^The source code is available at https://github.com/fassmeyer/labeling-situations.

^7^Recall that we use auxiliary labels in (Seq)LabelVAE to enforce sensible groupings.

^8^Opta Analytics Pro Forum, 2019 London https://www.youtube.com/watch?v=H1iho17lnoI.
